# rBPI_21_ Promotes Lipopolysaccharide Aggregation and Exerts Its Antimicrobial Effects by (Hemi)fusion of PG-Containing Membranes

**DOI:** 10.1371/journal.pone.0008385

**Published:** 2009-12-22

**Authors:** Marco M. Domingues, Miguel A. R. B. Castanho, Nuno C. Santos

**Affiliations:** Instituto de Medicina Molecular, Faculdade de Medicina da Universidade de Lisboa, Lisboa, Portugal; Charité-Universitätsmedizin Berlin, Germany

## Abstract

Antimicrobial peptides (AMPs) are important potential alternatives to conventional therapies against bacterial infections. rBPI_21_ is a 21 kDa peptide based on the N-terminal region of the neutrophil bactericidal/permeability-increasing protein (BPI). This AMP possesses highly selective bactericidal effects on Gram-negative bacteria and have affinity for lipopolysaccharide (LPS) which is believed to be at the origin of its neutralizing effect of the LPS segregated into the bloodstream. We aim at understanding the molecular bases of rBPI_21_ bactericidal and LPS neutralization actions, using biomembrane model systems. Using dynamic light scattering spectroscopy we demonstrate that rBPI_21_ promotes aggregation of negatively charged large unilamellar vesicles (LUV), even in the absence of LPS, and LPS aggregates, while for zwitterionic phosphatidylcholine (POPC) LUV the size remains unchanged. The peptide also promotes the fusion (or hemifusion) of membranes containing phosphatidylglycerol (POPG). The aggregation and fusion of negatively charged LUV are peptide concentration-dependent until massive aggregation is reached, followed by sample flocculation/precipitation. Concomitantly, there is a progressive change in the zeta-potential of the LUV systems and LPS aggregates. LUV systems composed of phosphatidylglycerol (POPG) and lipid mixtures with POPG have higher zeta-potential variations than in the absence of POPG. The interaction of rBPI_21_ with lipid vesicles is followed by leakage, with higher effect in POPG-containing membranes. LPS aggregation can be related with a decreased toxicity, possibly by facilitating its clearance by macrophage phagocytosis and/or blocking of LPS specific receptor recognition. Our data indicate that rBPI_21_ mechanism of action at the molecular level involves the interaction with the LPS of the outer membrane of Gram-negative bacteria, followed by internalization and leakage induction through the (hemi)fusion of the bacterial outer and inner membranes, both enriched in phosphatidylglycerol.

## Introduction

The innate immune system acts as a first line in the defense of many organisms against pathogens [1]. Antimicrobial peptides (AMPs) are part of the innate immune system of several organisms and usually have a broad spectrum of action against pathogens. During infection they are produced by the cells of the innate immune system and exert their effects on Gram-negative and Gram-positive bacteria, as well as on fungi, viruses and parasites [2]. AMPs have defined properties that ensure selective interaction with microbial plasma membranes [2]–[5]. Usually they are hydrophobic and have a positive net charge. Gram-negative bacteria have two membrane bilayers, the asymmetric outer membrane, mainly composed of lipopolysaccharide (LPS) in the outer leaflet and phospholipids in the inner leaflet, and the inner bilayer, composed by phospholipids [6]. The LPS molecules are very compact due to the salt bridge formed by the presence of Mg^2+^ and Ca^2+^ ions. This maintains an impermeable barrier to certain antibiotics and contributes to bacterial resistance.

LPS possess three distinct parts in their constitution: 1) a hydrophobic moiety, also denominated as lipid A (the biological active part [7]); 2) an oligosaccharide core attached to the lipid A via ketodeoxyoctonic acid (KDO); and 3) repeating saccharide units with different sizes depending on the bacterial species [6], [7]. During infection, bacteria release LPS from their membranes into the bloodstream of the host. Also, the LPS concentration in the bloodstream increases by the action of some antibiotics [8]. LPS interacts preferentially with monocytes/macrophages, promoting the release of several cytokines [9], [10]. The overproduction of these cytokines has harmful pathophysiological effects on the host, such as septicemia and lethal septic shock. Thus, it is necessary to find new antibiotics that exert cytotoxic effects and, at the same time, possess LPS-neutralization properties.

Human neutrophils have many anti-infective components in their granules. Bactericidal/permeability-increasing protein (BPI) is a 55-kDa protein located in primary granules of neutrophils [11], [12]. This protein acts selectively against Gram-negative bacteria where it exerts: 1) cytotoxic effects by membrane damage; 2) neutralization of the lipopolysaccharide endotoxin and; 3) opsonisation of bacteria, becoming a target to phagocytic cells. BPI has a boomerang shaped structure composed of two domains [13], [14]: whereas the cationic N-terminal region of BPI has antibacterial and LPS-neutralizing properties, the anionic C-terminal is required for opsonic activity [15], [16]. The 21 kDa recombinant N-terminal fragment of BPI (rBPI_21_), corresponding to the first 193 amino acids of BPI (except for the point mutation C132A [17]), also retains the bactericidal and neutralizing activity of the BPI [18], [19]. This fragment, presently on phase III clinical trials, was shown to be a promising agent against meningitis and septic shock [20]. Some studies of the interaction between rBPI_21_ and membranes have been reported [21]–[23]. However, previous studies have not focused on the membrane damage/alteration that occurs as a consequence of the interaction and its lipid selectivity.

In this paper we aim to address the question how the LPS molecules, segregated from the bacterial membrane, are neutralized, based on the *in vitro* interaction with rBPI_21_. Another issue is to understand the selectivity of rBPI_21_ membrane effects, to unravel the role of phospholipids and LPS on the increase of bacterial permeability.

## Methods

Phosphate buffer saline (20 mM sodium phosphate, 150 mM NaCl) pH 7.4 was used in all the measurements, except when indicated otherwise.

### Large Unilamellar Vesicles and Lipopolysaccharide Aggregates Preparation

Large unilamellar vesicles (LUV) with ∼100 nm of diameter were obtained by extrusion of multilamellar vesicles, as described elsewhere [24]. 1-palmitoyl-2-oleoyl-*sn*-glycero-3-phosphocholine (POPC) and 1-palmitoyl-2-oleoyl-sn-glycero-3-phospho-(1′-sn-glycerol) (POPG) were obtained from Avanti Polar Lipids (Alabaster, AL), while LPS (*Escherichia coli* O26:B6) was from Sigma (St. Louis, MO). The LUV studied were zwitterionic (pure POPC) or anionic (POPC∶LPS 80∶20 w∶w, POPC∶POPG 80∶20, POPC∶POPG 55∶45, pure POPG and POPC∶POPG ∶LPS 60∶20 ∶20). For lipid film formation with LPS, the LPS molecules were dissolved in chloform:methanol (2∶1) and the solution was extensively vortexed and bath sonicated at 40°C during 15 min. Stock solutions of LPS aggregates were bath sonicated at 40°C during 20 min. The stock solution was kept at 4°C overnight before measurements.

### Dynamic Light Scattering and Zeta-Potential Measurements of Large Unilamellar Vesicles in the Presence of rBPI_21_

Dynamic light scattering experiments were carried out on a Malvern Zetasizer Nano ZS (Malvern, UK) with a backscattering detection at 173°, equipped with a He-Ne laser (λ = 632.8 nm), at 37°C, using disposable polystyrene cells. The LUV suspensions were diluted to desired final concentration and then filtered using a syringe filter with 0.45 µm pore size (Whatman, Florham Park, NJ). Lipid concentration was kept constant at 50 µM and the rBPI_21_ (99% pure, kind gift from XOMA, Berkeley, CA) concentration varied from 24 nM to 238 nM. The samples were left equilibrating for 30 min at 37°C. Normalized intensity autocorrelation functions were analyzed using the CONTIN method [25], [26], yielding a distribution of diffusion coefficients (*D*). The measured *D* is used for the calculation of the hydrodynamic diameter (*D_H_*) through the Stokes-Einstein relationship:
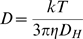
(1)


where *k* is the Boltzmann constant, T the absolute temperature, and *η* the medium viscosity. A set of 15 measurements (∼13 runs each) for the liposomes in the absence and presence of each different rBPI_21_ concentration were conducted to calculate de *D_H_* value. The *D_H_* of the sample was obtained from the peak with the highest scattered light intensity (i.e., the mode) in light scattering intensity distributions.

The zeta-potential (*ζ*) of the liposomes was determined at 37°C from the mean of 15 measurements (120 runs each), in the absence and presence of different rBPI_21_ concentrations, by phase analysis light scattering (PALS) in the previously indicated apparatus, using disposable zeta cells with gold electrodes. Values of viscosity and refractive index were set at 0.8872 cP and 1.330, respectively. Lipid concentrations were fixed at 200 µM in order to acquire high enough count rates. The electrophoretic mobility obtained was used for the zeta-potential calculation through the Smoluchowski equation:
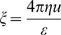
(2)


where *u* is the electrophoretic mobility, *η* the viscosity of the solvent and *ε* its dielectric constant.

### Dynamic Light Scattering and Measurement of Zeta-Potential of Lipopolysaccharide Aggregates in the Presence of rBPI_21_

The LPS aggregates were diluted to 300 µg.mL^−1^ (corresponding to 130 µM, taking into account the molecular weight of 2500 Da of deep rough LPS from *Salmonella* Re595 [27]) in a syringe and filtered through Whatman nylon filter with a pore diameter of 0.45 µm (Florham Park, NJ). rBPI_21_ concentration varied from 0.36 to 11.4 µM. The conditions used above for the LUV were applied to the measurements with LPS aggregates. A set of 15 measurements (∼13 runs each) for the LPS aggregates in the absence and presence of each different rBPI_21_ concentration were conducted to calculate de *D_H_* value. For the zeta potential measurements, the LPS aggregates were diluted in 100 mM Tris/HCl buffer with 2 mM CsCl, at pH 7.4, which yields more reproducible values. Zeta-potential was calculated from the mean of 15 measurements (120 runs each).

### LUV Lipid Mixing Assay in the Presence of rBPI_21_

Peptide-induced lipid mixing in vesicles was measured by Förster resonance energy transfer (FRET). This assay is based on the decrease in resonance energy transfer between two membrane probes, dipalmitoylphosphatidylehtanolamine-sulforhodamine B (RhB-PE) and 1,2-dihexadecanoyl-sn-glycero-3-phospho[N-4-nitrobenz-2-oxa-1,3-diazolyl]ethanolamine (NBD-PE), both from Sigma (St. Louis, MO), when the lipids of the vesicles labeled with both probes are allowed to mix with lipids from unlabelled vesicles. The concentration of each of the fluorescent probes within the pre-fusion liposome membrane was 0.6 mol %. LUV were prepared as described above. Labeled and unlabeled vesicles in a proportion of 1∶4, respectively, were placed in fluorescence cuvette at a total final lipid concentration of 100 µM, at 25°C, under constant stirring. The fluorescence was measured using a Varian Cary Eclipse fluorescence spectrophotometer (Mulgrave, Australia), with excitation at 470 nm and emission recorded between 500 and 650 nm. Excitation and emission slits were set to 10 nm. Phospholipid mixing was quantified on a percentage basis according to [28]:
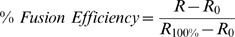
(3)


where *R* is the value of the ratio between the fluorescence intensity with emission at 530 nm and 588 nm, corresponding to the maximum fluorescence emission of NBD and RhB, respectively, obtained 10 min after peptide addition to a mixture containing liposomes having 0.6 mol% of each probe plus liposomes without any fluorescent probe. *R*_0_ is the ratio before peptide addition (constant during the evaluated time range), and *R*_100%_ the ratio after addition of Triton X-100 at a final concentration of 1% (v/v). For lipid mixing experiment, assays were conducted in triplicate.

### LUV Leakage Assay in the Presence of rBPI_21_ by Fluorescence Spectroscopy

Peptide-induced lipid vesicle leakage was also measured by fluorescence spectroscopy on a Varian Cary Eclipse fluorescence spectrophotometer (Mulgrave, Australia). In this assay we monitored the release of 5,(6)-carboxyfluorescein (CF), purchased from Sigma (St. Louis, MO), trapped in LUV. LUV were obtained as previously described, with the exception that the dried film was hydrated in 20 mM Tris-HCl with 150 mM NaCl, containing 100 mM CF (the pH was titrated to 7.4 with the addition of NaOH). Free CF was removed by passing the suspension through an Econo-Pac 10 DG column from Bio-Rad (Richmond, CA), where the vesicles are eluted with the void volume. Aliquots of eluted liposomes were used to determine the amount of phospholipid by the phosphorus assay [29]. Aliquots of the liposomal stock preparations (diluted to 10 µM) were incubated with various concentrations of the rBPI_21_ to be tested at 25°C, and fluorescence was recorded continuously for 30 min, under stirring (wavelength of excitation, λ_exc_ = 492 nm; wavelength of emission, λ_em_ = 517 nm). At the end of each experiment, total CF was determined after lysing the liposomes with 1% Triton X-100. The rates of CF leakage are expressed as the percentage of maximum trapped CF release, according to:
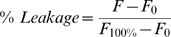
(4)


where *F* is the fluorescence intensity measured 20 min after peptide addition, *F*_0_ is the fluorescence intensity measured after 20 min without peptide addition, and *F*_100%_ is the fluorescence measured after disruption with Triton X-100. The fluorescence intensities were corrected for the dilution introduced by the addition of peptide and Triton X-100. For leakage experiment, assays were conducted at least in duplicate.

### Statistical Analysis

Unpaired Student's *t*-test was used for statistical analysis of intergroup comparison. Differences were considered statistically significant for *p*<0.05. Measurement reproducibility analysis yielded coefficients of variation <10% for the hydrodynamic diameter and zeta-potential experiments, and <1% for the fluorescence intensity measurements.

## Results and Discussion

### Dynamic Light Scattering of LPS Aggregates in the Presence of rBPI_21_

**Figure 1:**
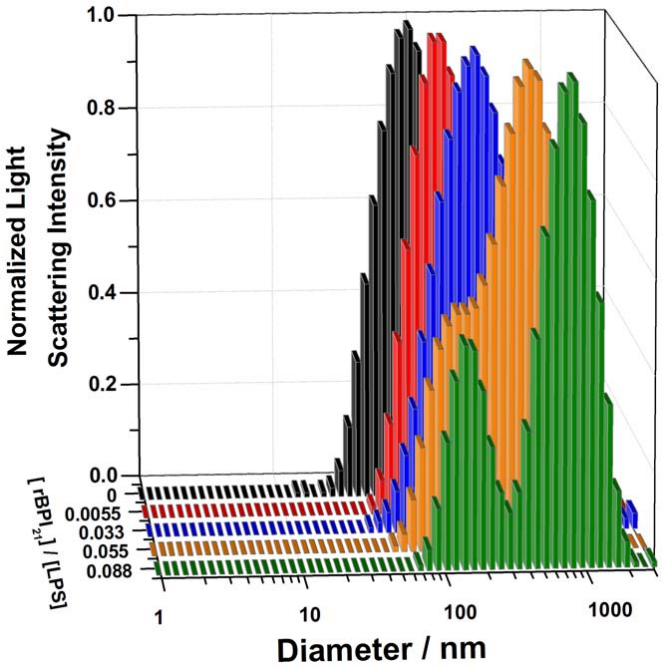
Size distributions of LPS aggregates in the presence of different concentration of rBPI_21_.

**Figure 2:**
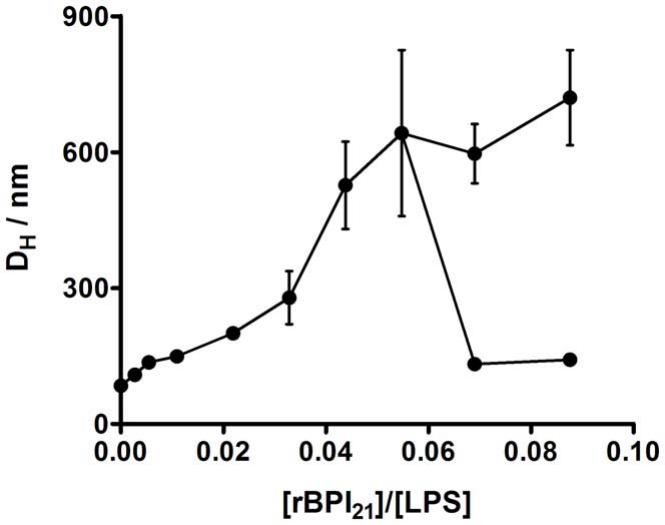
Aggregation of LPS promoted by rBPI_21_. Hydrodynamic diameter variation of LPS aggregates with increasing rBPI_21_ concentration. Bars represent the size range from three independent experiments.

To investigate the physical changes in the LPS aggregates, their size alterations in the presence of rBPI_21_ were studied by dynamic light scattering. rBPI_21_ promotes the aggregation of LPS aggregates in a concentration dependent manner (Figures 1 and 2). In solution, LPS aggregates have a size distribution centered at 84 nm (Figure 1). When peptide is added, larger particles are formed due to extensive aggregation. The polydispersity of the samples increases with the size increment. At rBPI_21_ concentrations above 9 µM, the sample size distribution becomes highly polydispersed with the onset of two independent populations. Higher rBPI_21_ concentrations promote massive aggregation and precipitation. This massive aggregation can contribute to reduce LPS toxicity, through the inhibition of the interaction of LPS with its receptor [30], [31] and by facilitating the phagocytosis of the aggregates by macrophages [32]. However, higher concentrations of the peptide are needed to promote significant aggregation of LPS when compared with the concentrations needed to cause equivalent effects in LUV (see below). The LPS used possesses a long chain of the saccharide portion, which hinders the interaction of the peptide with the negative charges of the LPS, due to the phosphate groups on lipid A. Thus, a higher peptide concentration seems to be needed to overcome the barrier created by the saccharide portion in order to promote aggregation.

### Zeta-Potential of LPS Aggregates in the Presence of rBPI_21_

**Figure 3:**
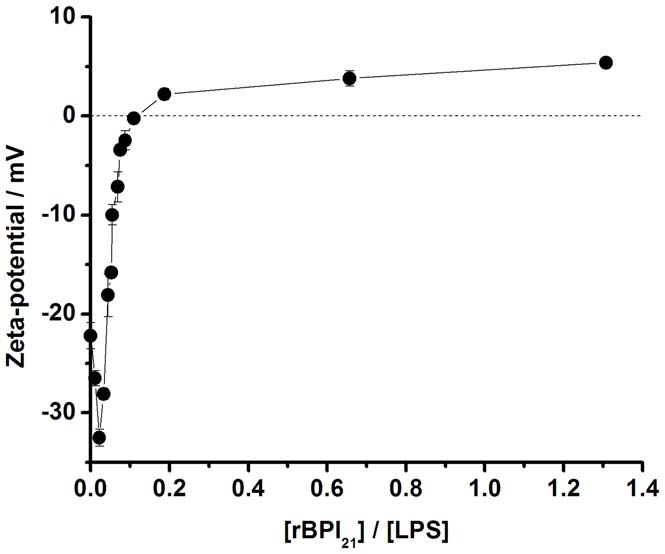
Zeta-potential for LPS aggregates in the presence of rBPI_21_. Bars represent the zeta-potential range from two independent experiments.

In order to evaluate if the peptide interacts with LPS aggregates by electrostatic forces, a detailed analysis of the interaction between rBPI_21_ and LPS was obtained from the electrophoretic mobility. Figure 3 shows that the LPS aggregates in the absence of the peptide possess a zeta-potential of −22 mV. Increasing concentrations of peptide promote the neutralization of the negative charge and even an overcompensation of the LPS aggregates charge at higher rBPI_21_ concentrations, which indicates an entrapment of the peptide beyond electrostatic equivalence.

### Dynamic Light Scattering of Liposomes in the Presence of rBPI_21_

**Figure 4:**
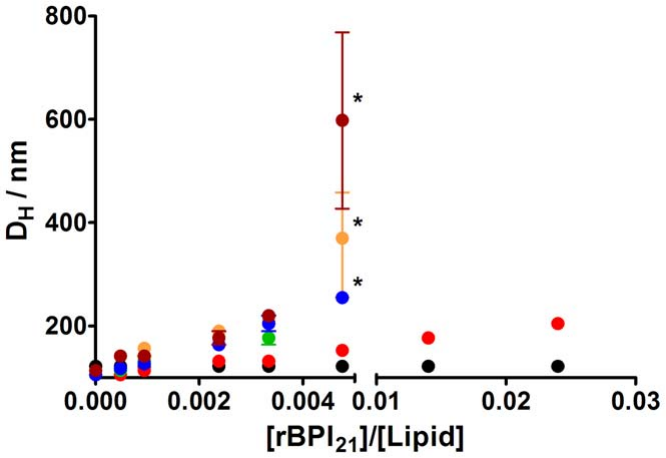
Aggregation of LUV caused by rBPI_21_. Hydrodynamic diameter variation of lipid vesicles with increasing rBPI_21_ concentration. Bars represents the size range from three independent experiments. Larger ranges are intrinsically associated to vesicles flocculation/precipitation (marked *). POPC (black circles); POPG (blue circles); POPC∶POPG 55∶45 (orange circles); POPC∶POPG 80∶20 (green circles); POPC∶LPS 80∶20 (red circles); POPC∶POPG∶LPS 60∶20∶20 (brown circles). The lipid concentration was kept constant at 50 µM. The differences in hydrodynamic size for [rBPI_21_]/[Lipid] = 0.0048 were statistically significant for the following data pairs: POPC/POPC∶POPG (80∶20), POPC/POPC∶POPG (55∶45), POPC/POPG, POPC/POPC∶POPG∶LPS (60∶20∶20), POPC∶LPS (80∶20)/POPC∶POPG (80∶20), POPC∶LPS (80∶20)/POPC∶POPG (55∶45), POPC∶LPS (80∶20)/POPG and POPC∶LPS (80∶20)/POPC∶POPG∶LPS (60∶20∶20).

To evaluate the perturbation in specific membrane compositions, physical changes in biomembrane model systems promoted by rBPI_21_ were assessed by dynamic light scattering. The size distributions obtained for the LUV show a low polydispersity, with a 106 nm average hydrodynamic diameter for all lipids and lipid mixtures, except for pure POPC LUV, which have a 122 nm average diameter (Figure 4). Upon addition of rBPI_21_, the hydrodynamic size of the LUV containing negatively charged LPS or POPG (bacterial membrane mimetic systems) increases. These larger entities correspond to the aggregation and/or fusion of the liposomes caused by the peptide. On the other hand, for zwitterionic membranes made of POPC (mammalian membrane mimetic system) the size does not change, even at larger rBPI_21_ concentrations (Figure 4). At peptide to lipid ratios higher than 0.005, the size of the lipid vesicles containing negatively charged POPG increases dramatically, leading to lipid vesicle aggregation, increased turbidity and, finally, precipitation. The peptide effect is more pronounced on the vesicles with POPG when compared with POPC and POPC∶LPS (80∶20). This result can be explained by the loss of electrostatic attraction by POPC and in POPC∶LPS membranes due to the compact structure of LPS and also by the size of the saccharide portion, which forms a steric barrier for aggregation [33]. However, for membranes of POPC with both POPG and LPS (20% each) the result obtained is not significantly different of the obtained for membranes composed of POPC and POPG or pure POPG (Figure 4). Nomura *et al.* showed that in membranes composed of negatively charged phospholipids, the incorporation of deep rough mutant LPS (ReLPS) is severely reduced [34]. Furthermore, the membrane-incorporated ReLPS forms LPS or LPS/phospholipid clusters in certain regions of the membrane. This can result from the electrostatic repulsion between POPG and ReLPS. The aggregated ReLPS, due to its compact nature and to the steric obstacle caused by the saccharide portion, has an impaired interaction with the peptide. Thus, the peptide may accumulate at the level of the accessible POPG headgroups and promote the vesicle aggregation/fusion, as occurs for POPC∶POPG mixtures and POPG liposomes. The same result was also obtained by Allende and McIntosh, by showing that the addition of bacterial phospholipids to deep rough LPS membranes increases the susceptibility to melittin [35].

### Zeta-Potential of Liposomes in the Presence of rBPI_21_

**Figure 5:**
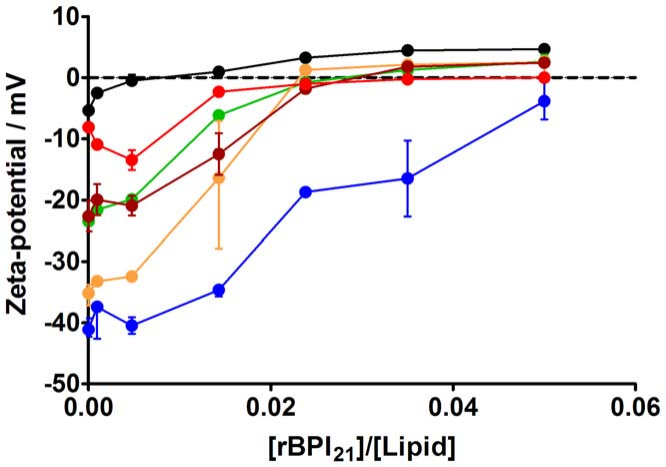
Zeta-potential for membrane model systems in the presence of rBPI_21_. Bars represent the zeta-potential range from at least two independent experiments. POPC (black circles); POPG (blue circles); POPC∶POPG 55∶45 (orange circles); POPC∶POPG 80∶20 (green circles); POPC∶LPS 80∶20 (red circles); POPC∶POPG∶LPS 60∶20∶20 (brown circles). The lipid concentration was kept constant at 200 µM. The differences in potential-zeta for [rBPI_21_]/[Lipid]  = 0.015 were statistically significant for the following data pairs: POPC/POPC∶LPS (80∶20), POPC/POPC∶POPG (80∶20), POPC/POPC∶POPG (55∶45), POPC/POPG, POPC/POPC∶POPG∶LPS (60∶20∶20), POPC∶LPS (80∶20)/POPG; POPC∶POPG (80∶20)/POPG, POPC∶POPG (55∶45)/POPG and POPC∶POPG∶LPS (60∶20∶20)/POPG.

In order to analyze the contribution of the electrostatic force in peptide-biomembrane models interaction, zeta-potential measurements were done with membrane mimetic systems, as previously described in the Methods section. For POPC∶POPG biomembrane models with different proportions of POPG (0%, 20%, 45% and 100%) without the addition of the peptide, lipid vesicles present zeta-potential values of −5.3 mV, −23.5 mV, −35.2 mV and −41.1 mV, respectively (Figure 5). For biomembrane models of POPC∶LPS and POPC∶POPG ∶LPS the initial zeta-potential value are −7.7 mV and −25.2 mV, respectively. Those potentials increase toward positive values when the peptide is present, with different behaviour for the tested membrane mimetic systems. Zeta-potential can be used as a measure of sample stability. The general dividing line between stable and unstable suspensions is generally considered at ±30 mV. Particles with zeta-potentials more positive than +30 mV or more negative than −30 mV are normally considered stable, whereas the particles with zeta-potentials between −30 mV and +30 mV have tendency to flocculate [36]. At the limit peptide to lipid ratio for dynamic light scattering measurements of 0.005, the zeta-potential of the lipid vesicles were within the positive instability region, except for membrane lipid systems composed of POPC∶POPG 55∶45 and pure POPG (Figure 5). As a consequence, the aggregation/flocculation of the systems composed of POPC∶POPG or pure POPG cannot be explained only by the electrostatic interactions. A bridging phenomenon seems to motivate the aggregation, by the interaction of the rBPI_21_ with several liposomes due to high number of positive amino acids on the peptide [37]. The unfolding of the peptide when interacting with membranes exposes some of the positive charges toward the medium, promoting the bridging between liposomes [21]. At higher peptide to lipid ratios an overcompensation of the potential is observed. This means that the peptide not only adsorbs on the surface of the vesicles, neutralizing its charge, but also inserts on the membrane, due to hydrophobic interactions [21]. Another aspect that should be referred is the interaction of the peptide with neutral POPC vesicles, observed by the zeta-potential measurements, while the size remained unchanged, as seen by dynamic light scattering. Apparently, the interaction of the peptide with neutral liposomes is weaker, when compared to the negatively charge membranes, and must be limited to the surface of the bilayer, making this interaction difficult to detect by other methods [21]. rBPI_21_ interaction with pure POPC bilayers may be explained by the repulsion of the positive charged end group of the choline towards the aqueous phase, due to the high density of the positive amino acids in rBPI_21_, exposing the hydrophobic region of the lipid for the interaction with the peptide [38], [39]. Despite the lower aggregation state of the POPC∶LPS in the presence of rBPI_21_, the peptide is able to interact with negative charges of the LPS lipid A. The result demonstrates that rBPI_21_ penetrates into the saccharide region to interact with the lipid A. On the other hand, for systems composed of POPC∶POPG ∶LPS, in the presence of the peptide the zeta-potential trend increases in a similar way as the POPC with 20% of POPG. This behavior indicates a preference of the peptide for POPG due to its accessible polar head.

### Fusion of Liposomes Promoted by rBPI_21_

**Figure 6:**
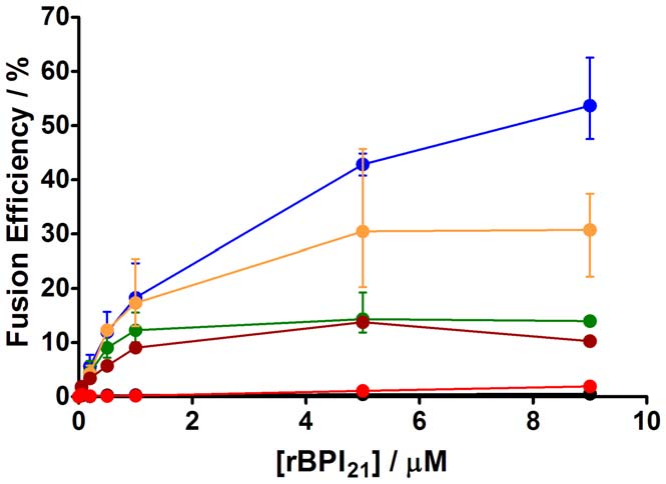
Fusion or hemifusion of membrane model systems promoted by rBPI_21_. LUV labeled with 0.6% mol of RhB-PE and NBD-PE were mixed with unlabeled liposomes to a final proportion of 1∶4 and the energy transfer assay was evaluated in the presence of rBPI_21_ after 10 min of incubation. Bars represent the range from three independent experiments. POPC (black circles); POPG (blue circles); POPC∶POPG 55∶45 (orange circles); POPC∶POPG 80∶20 (green circles); POPC∶LPS 80∶20 (red circles); POPC∶POPG∶LPS 60∶20∶20 (brown circles). The differences in (hemi)fusion percentage for rBPI_21_ 9 µM were statistically significant between all data pairs, except POPC/POPC∶LPS (80∶20).

As aggregation occurs, there is a significant fusogenic activity promoted by rBPI_21_, mainly on negatively charged liposomes (Figure 6). The highest membrane fusion efficiency was observed for pure POPG liposomes. The fusion efficiency increases with the percentage of POPG content in the POPC∶POPG mixtures. There is no rBPI_21_-induced fusion of pure POPC liposomes. At variance with what could be expected, when the negatively charged molecules of LPS are added to liposomes the fusion efficiency does not change. Therefore, rBPI_21_-induced fusion is limited to POPG-containing membranes. The fusion behavior of membranes composed of POPC with POPG and LPS (20% each) is similar to liposomes made of POPC with 20% of POPG. The results reinforce the idea that the peptide exerts higher membrane destabilization in the presence of POPG and that the presence of LPS does not cause an increase of the destabilization. This can be due to its saccharide region, acting as a steric barrier for liposome-liposome interaction. It is important to bear in mind that membrane hemifusion can also contribute to the parameter here indicated as fusion efficiency.

### rBPI_21_-Induced Carboxyfluorescein Leakage from Liposomes

**Figure 7:**
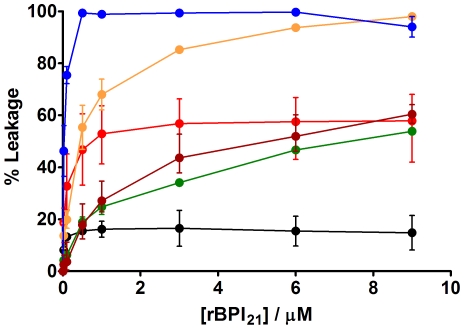
rBPI_21_-induced membrane leakage. Carboxyfluorescein fluorescence increase measured 20 min after rBPI_21_ addition in POPC (black circles), POPC∶LPS 80∶20 (red circles), POPC∶POPG 80∶20 (green circles), POPC∶POPG 55∶45 (orange circles), POPC∶POPG∶LPS 60∶20∶20 (brown circles) and POPG (blue circles) liposomes. Bars represent the range from at least two independent experiments. The differences in leakage percentages for rBPI_21_ 9 µM were statistically significant for the following data pairs: POPC/POPC∶LPS (80∶20), POPC/POPC∶POPG (80∶20), POPC/POPC∶POPG (55∶45), POPC/POPG, POPC/POPC∶POPG∶LPS (60∶20∶20), POPC∶LPS (80∶20)/POPG∶POPG (55∶45), POPC∶LPS (80∶20)/POPG, POPC∶POPG (80∶20)/POPC∶POPG (55∶45), POPC∶POPG (80∶20)/POPG, POPC∶POPG (55∶45)/POPC∶POPG∶LPS (60∶20∶20) and POPC∶POPG∶LPS (60∶20∶20)/POPG.

To complete the evaluation of the interaction of rBPI_21_ with biomembrane model systems, the ability of the peptide to induce the leakage of carboxyfluorescein from liposomes was measured (Figure 7). The peptide exerts its higher effect on membranes composed of negatively charged lipid. However, the effect is more pronounced in membranes with higher content of POPG. As seen before the electrostatic force acts as a main force for peptide interaction, but there are other contributions for the effect.

### Conclusion

We have shown that rBPI_21_, at 37°C, interact with negatively charged LPS aggregates promoting their extensive aggregation. This action can be important for the clearance of LPS from the bloodstream, promoting its phagocytosis by macrophages. The aggregation of LPS promoted by rBPI_21_ can also interfere with the interaction of LPS with its receptor [30], [31]. Thus, rBPI_21_ plays a role against sepsis by neutralizing LPS toxicity.

Looking at the results obtained with biomembrane models systems, one can conclude that the membrane destabilization effects are only partially charge-dependent. The electrostatic force plays a key role for the bactericidal action of the peptide as seen by surface charge studies. When POPG is present both aggregation and fusion occurs. rBPI_21_-induced membrane leakage is also more pronounced for POPG-containing membranes. These results explain the specificity of the peptide for bacterial membranes, in comparison with mammalian membranes. For liposomes containing LPS, the membrane destabilization is more pronounced when POPG is present. This result is consistent with the higher susceptibility of deep rough bacteria when compared with rough and smooth strains [40]. Deep rough bacteria have higher phospholipid to LPS ratios in the outer leaflet of the outer membranes, which contributes to a higher interaction with other molecules. This can explain the higher susceptibility of deep rough bacteria to antibiotics. In the absence of POPG, membranes of POPC∶LPS behave like POPC membranes in terms of rBPI_21_-induced membrane fusion and extensive aggregation. The saccharide region of LPS may act as a barrier against fusion and aggregation, specially for strains with LPS with longer saccharide components. However, leakage also occurs when LPS is present.

**Figure 8:**
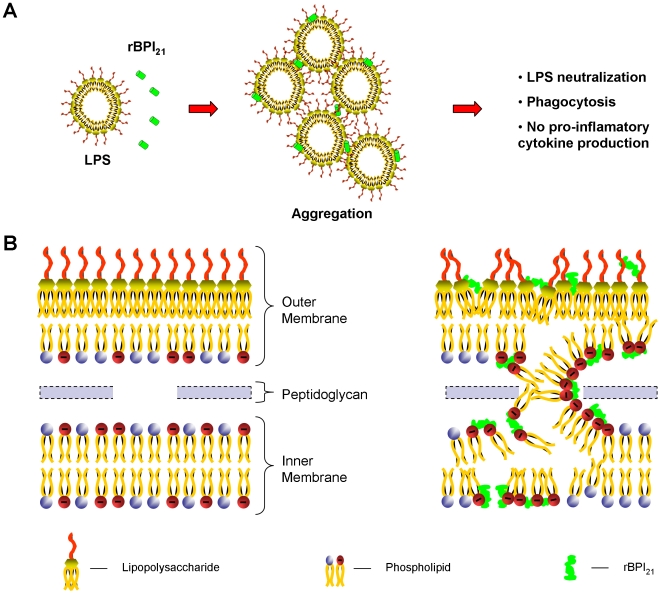
Schematic representation of the proposed mechanism of action of rBPI_21_ protein. A) The protein interacts with LPS aggregates promoting their aggregation, which allow LPS phagocytosis by innate immune cells and reduce LPS availability for its receptor. B) rBPI_21_ interacts electrostatically with the LPS of the outer leaflet of outer membrane. This interaction disrupts the higher tight bond of the LPS, which allow the peptide insertion and translocation to the outer membrane inner leaflet and to the intermembrane space. rBPI_21_ induces the fusion (or hemifusion) of the inner leaflet of the outer membrane and the inner membrane (both rich in PG), following a process similar to the occurring at the Bayer junctions [41]. These membrane fusion events promote an increased membrane permeability, culminating with the leakage of the bacterial content.

Our findings show that the peptide is able to interact with LPS of the outer membrane and could translocate to the intermembrane space, enabling the interaction with the inner membrane, where it exerts higher antimicrobial activity. Based on the results obtained, a schematic representation of the mechanism of action of rBPI_21_ is proposed (Figure 8). For LPS aggregates in the bloodstream, rBIP_21_ interacts electrostatically with LPS promoting their aggregation and neutralizing its toxicity (Figure 8A). Upon interaction with Gram-negative bacteria, rBPI_21_ is able to bind to the outer leaflet of the outer membrane, enriched in LPS, by electrostatic forces. Afterwards, rBPI_21_ inserts into the LPS-rich membrane [21] and moves to the space between the two bacterial membranes. As both the inner leaflet of the outer membrane and all the inner membrane are rich in PG, rBPI_21_ is able to induce the binding between the two bacterial membranes and their fusion (or hemifusion), following a process similar to what happens in the Bayer junctions [41]. Based on this model and on our results we propose that the fusion or hemifusion of the two bacterial membranes, rich in PG, are the real cause of the changes in permeability and leakage of bacteria content reported for rBPI_21_ and BPI (Figure 8B), instead of the simple interaction with LPS, fully explaining at the molecular level both the mechanism of microbicide action of rBPI_21_ and the naming of BPI as “bactericidal/permeability-increasing protein”.
